# The impact of healthy community environment and sleep quality on the physical health of older adults: evidence from CGSS2021

**DOI:** 10.3389/fpsyg.2025.1556043

**Published:** 2025-07-30

**Authors:** Xiuchao Wei, Wenwu Dai, Yue Xie

**Affiliations:** ^1^No.1 High School of Qinhuangdao, Qinhuangdao, China; ^2^School of Humanities and Social Sciences, Beijing Forestry University, Beijing, China

**Keywords:** healthy community environment, sleep quality, subjective and objective physical health, older adults, CGSS2021

## Abstract

As the aging population expands, the proportion of older adults facing physical health challenges is expected to increase substantially. A fundamental question arises as to whether healthy community environment can effectively enhance the physical health of the elderly. This study, based on the 2021 Chinese General Social Survey, included a total of 696 participants aged 65 and above. Our study aimed to investigate the impact of healthy environment, healthy service, and healthy neighborhood on physical health of the elderly, as well as the mediating effects of sleep quality in these relationships. The results are as follows: (1) There were significant positive correlations between healthy environment, healthy service, healthy neighborhood, and sleep quality in pairwise relationships. There was also a significant positive correlation between healthy environment and objective physical health. Similarly, healthy services and healthy neighborhood each had significant positive correlations with subjective physical health, and sleep quality exhibited significant positive correlations with both subjective and objective physical health. (2) After controlling for variables such as gender, age, and perceived socioeconomic status, sleep quality played a mediating role in the relationship between the healthy community geographical environment and physical health of the elderly. The research findings provide empirical evidence for enhancing the physical health of the elderly from the perspective of building a healthy community.

## 1 Introduction

As of the end of 2022, the population of individuals aged 60 and above in China reached 280.04 million, accounting for 19.8% of the total population. Among them, 209.78 million were aged 65 and above, comprising 14.9% of the national population. These statistics underscore that China is undergoing a rapid demographic shift toward population aging, which has become a critical societal concern both domestically and globally (Yang et al., 2025). As the aging population expands, the proportion of older adults facing physical health challenges is expected to increase substantially, mainly due to age-related physiological decline (Liljas et al., 2017). Poor physical health not only adversely affects cognitive functioning (Kim et al., 2005), daily activity performance, and psychological well-being (Cappeliez et al., 2004), but also contributes to elevated mortality risk (Michael et al., 2005). Moreover, it significantly amplifies the demand for geriatric healthcare services, placing unprecedented pressure on the healthcare system (Bai et al., 2021; Luo et al., 2024). Therefore, identifying the determinants of physical health in older adults is of paramount importance for both academic inquiry and public policy planning.

Physical health refers to the overall functioning of the body, including the condition of its organs and physiological systems (Li and Zhou, 2012). In current gerontological research, physical health is typically assessed using either subjective or objective measures. Subjective assessments rely on older adults’ self-evaluations of their physical condition, and have been shown to be reliable predictors of various health outcomes (Jiao et al., 2018; Jylhä, 2009). In contrast, objective assessments are based on clinically diagnosed chronic conditions, which provide medically grounded indicators of physical impairment (Shan et al., 2016; Fan et al., 2019; Fan et al., 2022). Both approaches capture different facets of physical health, and incorporating both offers a more comprehensive understanding of aging-related health status. The ecological framework of aging highlights the potential impact of environmental factors on the physical health and overall well-being of older individuals (Bronfenbrenner and Morris, 1998). Previous research has extensively explored the effects of micro-systems, such as marital and parent–child relationships, on the physical health of the elderly (Lu et al., 2023; Novak et al., 2023). However, investigations into the influence of the community, which represents a pivotal system, on the physical health of the elderly are still in their early stages. Communities represent the most significant spatial context in the lives of the elderly and play a vital role in shaping healthy lifestyle choices and ensuring the physical health of older adults (Song et al., 2024). From the perspective of user experience design (UXD), the effectiveness of community environments is largely determined by how older residents perceive and emotionally respond to their surroundings. Therefore, the design of community environments should prioritize user-centric principles (Moser, 2012). Moreover, UXD theory posits that user experience is shaped by multiple dimensions, such as usability, accessibility, and contextual appropriateness, among others, all of which play a crucial role in shaping health-related outcomes for older adults. However, there is currently no consensus among researchers regarding the criteria for evaluating healthy communities. Before delving into the exploration of which factors in healthy communities influence the physical health of the elderly, our study initially conducted a review and systematization of the evaluation criteria for healthy communities based on prior research.

**Figure 1:**
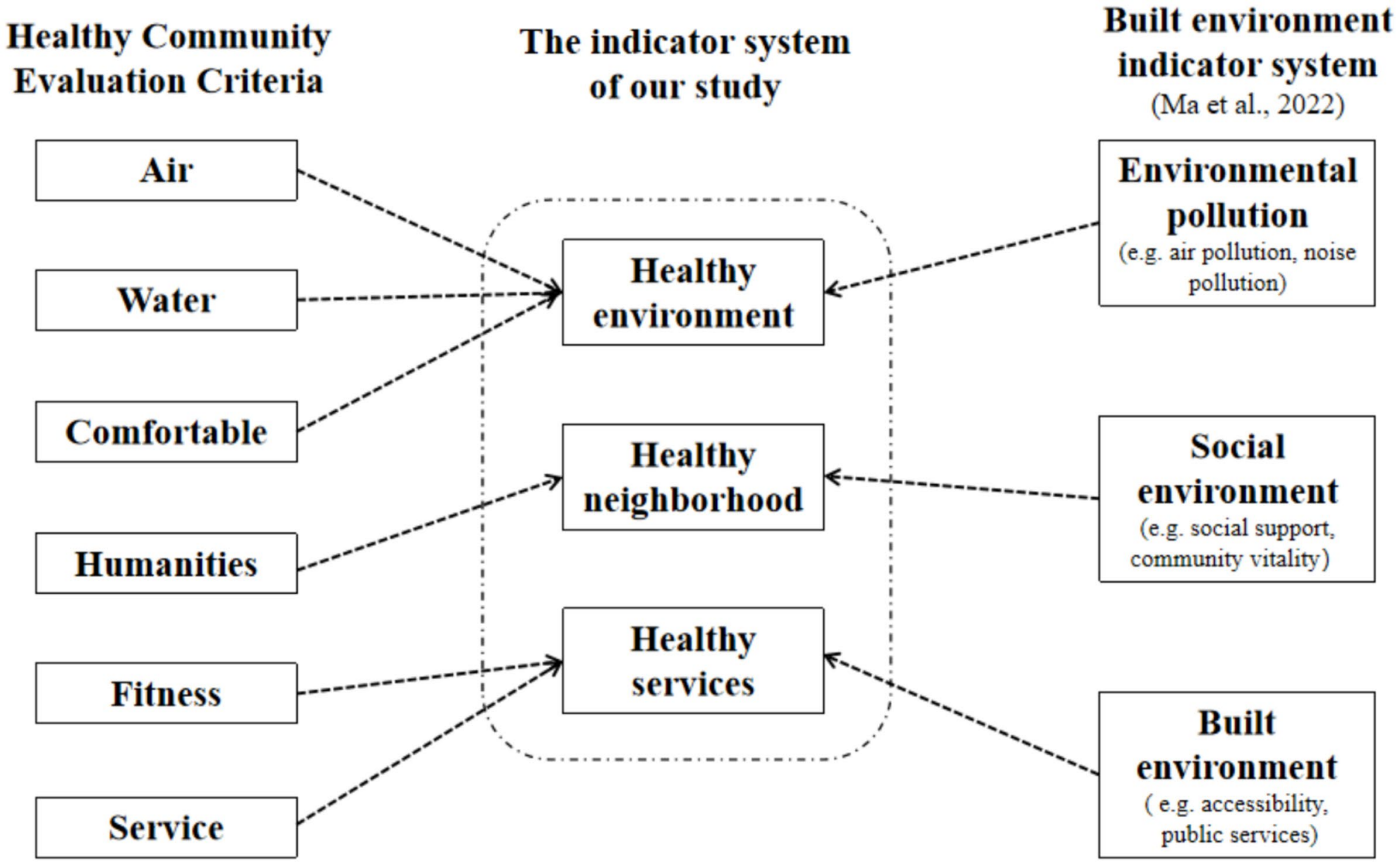
The assessment indicator system for healthy communities in our study. This figure illustrates how the indicator system developed in this study was constructed by integrating elements from two existing frameworks: the healthy community evaluation criteria (left) and the built environment indicator system proposed by Ma et al. (2022) (right). The three core dimensions—healthy environment, healthy neighborhood, and healthy services—were derived from overlapping and conceptually aligned indicators across both frameworks. Dotted arrows indicate the conceptual mapping from existing criteria to our study’s constructs.

In March 2020, China introduced its inaugural “Healthy Community Evaluation Criteria” (Zhong et al., 2022). These standards principally encompass a spectrum of assessment criteria across various domains. These domains encompass air quality, water quality, comfort, health facilities, humanity factors, and services. While these criteria span a diverse array of domains, these assessment standards primarily emphasize the influence of environmental factors on the health of residents, while the categorization of community services and psychological factors remains relatively vague. Meanwhile, Ma et al. (2022) primarily utilized three major indicator systems to analyze the community environment, encompassing the built environment (e.g., accessibility, public services), the social environment (e.g., regional deprivation, social support), and environmental pollution (e.g., air and noise pollution). In this study, we integrated Ma’s geographical environmental assessment framework with the “Healthy Community Evaluation Criteria,” resulting in the development of a Geographical Environmental Assessment Indicator System for Healthy Communities in our study (Figure 1). Healthy environment mainly entails residents’ subjective assessments of the level of environmental pollution in the community. Healthy services primarily involve residents’ subjective assessments of the availability of fresh food and the accessibility of public facilities in the community. Healthy neighborhood encompasses residents’ subjective evaluations of a sense of security, neighborly assistance, and neighborly concern within the community.

The healthy community geographical environment may have a close relationship with the physical health of the elderly. First, environmental pollution is a major risk factor for chronic diseases, and older adults are especially vulnerable to its effects. Studies have shown that air and water pollution significantly increase the incidence of chronic illnesses (Fan et al., 2022), while noise pollution—considered the second-largest threat to public health—can have irreversible impacts on physical function and is also linked to poorer subjective health perceptions (Münzel et al., 2014; Herrera and Cabrera-Barona, 2022). Recent studies have shifted toward multidimensional assessments of environmental quality. For example, Yuan et al. (2020) developed a pollution index integrating multiple indicators and found that although environmental pollution may not have strong short-term health effects, it contributes to declines in individuals’ subjective physical health. Second, the availability of health-related services within a community is positively associated with physical health outcomes. A qualitative study has indicated that the availability of built services within a community plays a crucial role in maintaining individual health (Addy et al., 2004). Several studies have demonstrated a connection between the accessibility of sports facilities and individual sports participation, which positively contributes to enhancing an individual’s physical health (Eriksson et al., 2012; Xue and Li, 2023). In contrast, residents in resource-deprived communities often face restricted opportunities for healthy living, which can lead to poorer physical health (Yang and South, 2020). Third, a healthy neighborhood fosters social cohesion and perceived safety, both of which play a critical role in maintaining physical health. Community cohesion has been shown to significantly predict self-rated health by reducing stress and enhancing psychological well-being (Bjornstrom et al., 2013). Strong neighborhood ties and perceived support can improve access to health information and services, indirectly promoting better physical health in older adults. Based on the above research findings, we propose the following hypotheses:

*H1A*: There is a significant correlation between the healthy environment and the physical health of the elderly.

*H1B*: There is a significant correlation between healthy services and the physical health of the elderly.

*H1C*: There is a significant correlation between healthy neighborhood and the physical health of the elderly.

Beyond the direct influence of the healthy community environment on physical health, sleep quality may serve as an important mediating mechanism. Prior research has shown that sleep quality is significantly associated with both objective and subjective aspects of physical health. Longitudinal studies from Canada and the UK have found that self-reported sleep duration and sleep satisfaction in older adults are associated with an increased risk of developing chronic illnesses (Nicholson et al., 2020; Sabia et al., 2022). Similar research findings have been validated in the elderly population in China: older adults with generally poorer sleep quality have a higher risk of comorbid chronic diseases compared to those with better sleep quality (Xia et al., 2023). Sleep quality can also significantly predict an individual**’**s subjective physical health. A study based on adults from a Texas community discovered that subjective sleep quality positively predicts subjective physical health (Hale et al., 2010). Based on a meta-analysis, self-reported sleep quality in older adults exhibits a moderate positive effect on their physical health (*r* = 0.35), while there is no significant correlation with objective sleep quality (Sella et al., 2023). Therefore, the present study focuses on the mediating role of subjective sleep quality, as it may better reflect individuals’ health perceptions in the context of healthy community environments.

The healthy community geographical environment is also associated with sleep quality of the elderly. Hale et al. (2013) proposed three pathways through which the community environment influences sleep quality, namely, ambient hazards and physical exposures, psycho-physiological pathways, and health behavior. The healthy community assessment system proposed in this study covers these three aspects and, to some extent, enriches these pathways, which suggests that the community geographical environment assessment indicators introduced in this study can also impact sleep quality. First, the healthy environment dimension plays a significant role in predicting sleep quality. Extensive research has demonstrated that exposure to air pollution negatively affects sleep by disrupting the central respiratory and nervous systems, and by increasing psychological stress responses (Cao et al., 2021). In contrast, natural sunlight exposure has been found to improve sleep outcomes by enhancing circadian rhythm regulation, which supports better sleep timing and quality (Harb et al., 2015; Burns et al., 2021). Additionally, noise pollution has shown long-term detrimental effects, with studies indicating that exposure can predict the occurrence of sleep disturbances even 5 years later (Beutel et al., 2020). These findings highlight the importance of a clean, quiet, and light-balanced environment for promoting healthy sleep, particularly among older adults. Furthermore, the predictive role of healthy services on sleep quality is significant. Communities equipped with more comprehensive infrastructure and services tend to yield residents with enhanced sleep quality (DeSantis et al., 2016). If a community can provide fresh fruits and vegetables to its residents, it influences their dietary choices to a certain extent. A healthy and well-balanced dietary structure, coupled with the consumption of nutritious foods, is associated with improved sleep quality (Godos et al., 2021). The proximity of sports facilities and services also motivates residents to engage in physical activities willingly. Active participation in physical exercise can influence sleep quality and serves as an effective means to enhance it. Lastly, healthy neighborhood has a significant predictive effect on sleep quality. Feelings of insecurity within a community can trigger short-term anxiety, fear, and despair (Hale et al., 2010). These emotions activate stress responses, prompting the release of stress hormones, ultimately increasing physiological arousal in the elderly and resulting in decreased sleep quality (Van Reeth et al., 2000). A study focused on African Americans has revealed significant correlations between community cohesion, neighborhood safety, and various sleep quality indicators, including sleep latency, sleep duration, and sleep efficiency (Nam et al., 2018). Based on the research findings mentioned above, we propose the following hypotheses:

*H2A*: Sleep quality serves as a mediator in the relationship between healthy environment and the physical health of older individuals.

*H2B*: Sleep quality serves as a mediator in the relationship between healthy services and the physical health of older individuals.

*H2C*: Sleep quality serves as a mediator in the relationship between healthy neighborhood and the physical health of older individuals.

**Figure 2:**
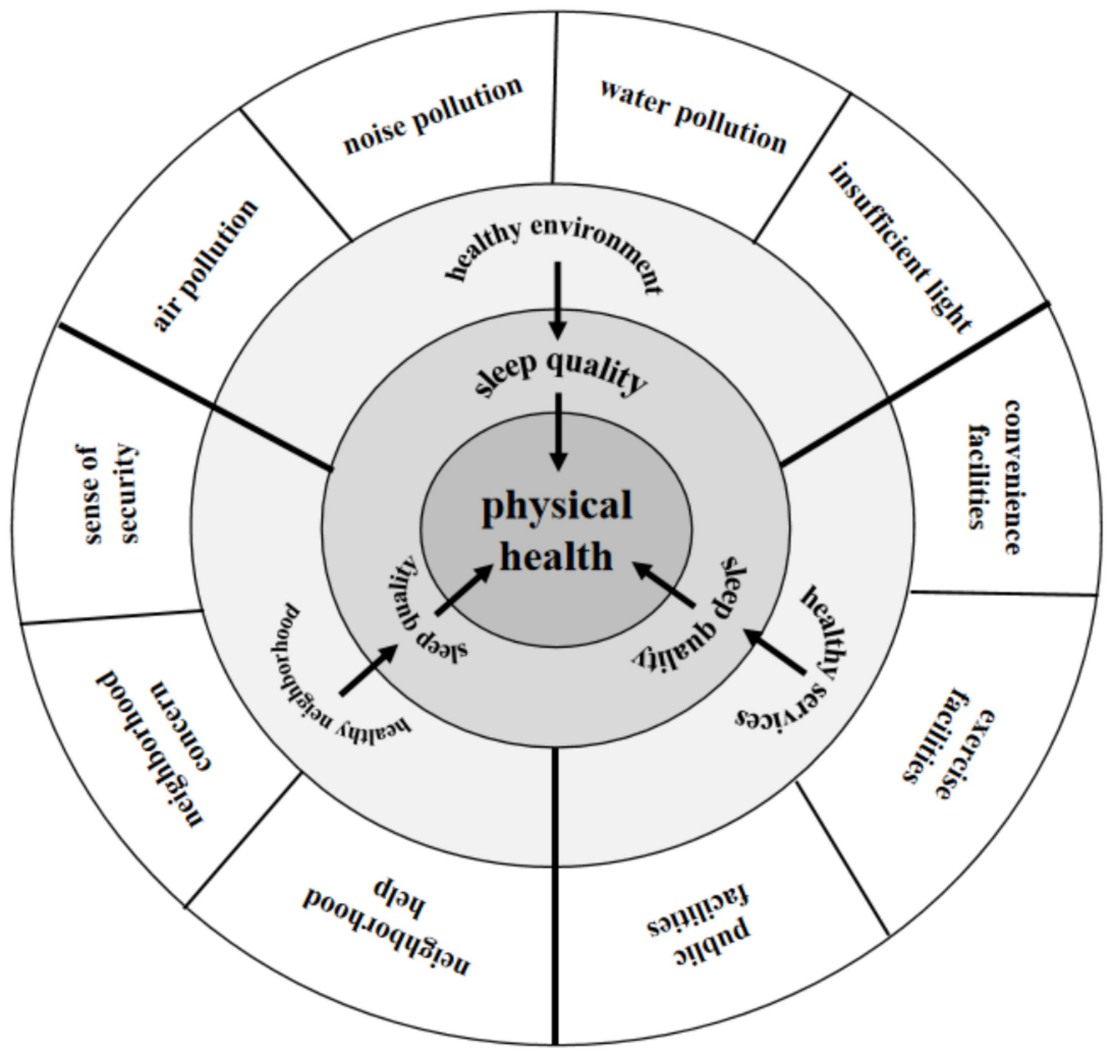
The model of the effect of healthy community geographical environment on the physical health of the elderly in this study. This figure illustrates a conceptual framework in which specific environmental indicators (outer layer) are grouped into three key dimensions: healthy environment, healthy services, and healthy neighborhood (middle layer). These dimensions influence older adults’ sleep quality (inner layer), which in turn predicts their physical health (center).

Based on the above literature analysis, although previous studies have explored the relationships among healthy community geographical environment, sleep quality, and the physical health, they still suffer from several limitations: (1) The study populations have typically been generalized, with a predominant focus on adults, and there has been limited attention given to individuals aged 65 and above; (2) Research on healthy community geographical environment has predominantly employed an integrative perspective. Hale et al. (2013) proposed three pathways—ambient hazards and physical exposures, psycho-physiological pathways, and health behavior—influencing sleep quality among older adults. However, this study relied on calculating total scores for community environmental features and categorizing community quality into high and low, which does not allow for the exploration of each pathway’s specific mechanisms influencing the sleep quality and the physical health of older individuals; (3) Studies on the physical health of older adults have predominantly used subjective or objective assessment methods. However, the factors affecting subjective and objective physical health may not be the same, and relying solely on one assessment method may not fully describe the factors that affect the physical health of the elderly. Addressing these limitations, our study narrows its focus to the elderly, constructs a healthy community geographical environment assessment system, and employs three different assessment dimensions to analyze the relationships between community geographical environment, sleep quality, and the subjective and objective physical health of older adults. Our study aims to identify the factors and mechanisms affecting the physical health of the elderly, as detailed in Figure 2.

## 2 Methods

### 2.1 Data source

The China General Social Survey (CGSS) is conducted by the National Survey Research Center at Renmin University of China.^1^ CGSS has been an ongoing, nationally representative survey project in China since 2003, collecting multidimensional information on various aspects of society, communities, households, and individuals. For the purposes of this research and data availability, we selected CGSS 2021 as the source of our study data. The 2021 wave of CGSS was conducted with ethical approval from the Institutional Review Board of Renmin University of China (Ethics Approval Number: RN2010040901), and all participants provided written informed consent prior to participation. The dataset used in this study was fully anonymized by the data provider prior to public release, ensuring the protection of personal privacy. All procedures complied with the ethical standards outlined in the Declaration of Helsinki.

In CGSS 2021, a total of 8,184 valid responses were collected across 19 provinces in China through the administration of core survey modules. In particular, specialized research modules were randomly assigned to selected sub-samples of respondents, covering topics such as health, physical activity, and the living environment. Based on our research objectives and the availability of relevant variables, we conducted a series of data preprocessing steps. First, we extracted all respondents with valid age information and selected those aged 65 years and above. We then retained only those individuals who had completed the relevant specialized modules required for our analysis, which focused on three core variables: physical health, sleep quality, and healthy community. Finally, we excluded cases with missing or invalid values on any key study variables. After applying these criteria, the final working sample consisted of 696 older adults, whose demographic distribution is presented in Table 1.

**Table 1:** Participants distribution and descriptive statistical results of variables.

Variable	N	Proportion (%)	Min	Max	M	SD
Dependent variable						
Subjective physical health	696		1	5	3.11	1.07
Objective physical health	696		0	8	6.89	1.24
Independent variable						
Healthy environment	696		1	4	3.22	0.58
Healthy service	696		1	5	3.66	0.81
Healthy neighborhood	696		1	5	4.16	0.64
Mediating variable						
Sleep quality	696		1	4	2.85	0.86
Control variables						
Gender	696		0	1		
Female	696	354				
Male	696	342				
Perceived socioeconomic status	696		1	5	3.67	0.97
Age	696		65	95	72.66	6.07

### 2.2 Variables and research tools

In this study, we investigated the physical health (incorporating subjective and objective components) as the dependent variables. We considered the healthy community geographical environment (comprising healthy environment, healthy service, and healthy neighborhood) as the independent variables, while sleep quality as the mediating variable. We also included three control variables. Detailed descriptive statistics can be found in Table 1, and the specific survey questions are outlined in Table 2.

**Table 2:** Research variables and measurement items.

Type	Variable	Items
Independent variables	Healthy environment	How severe is air pollution where you live?
		How severe is water pollution where you live?
		How severe is noise pollution where you live?
		How severe is the lack of light where you live?
	Healthy services	The place I live is suitable for physical activities (e.g., jogging, walking)
		Where I live, there are many fresh vegetables and fruits to choose from.
		Where I live, there are adequate public facilities (e.g., community centers, parks.)
	Healthy neighborhood	It’s safe where I live.
		Neighbors around me care about each other.
		Neighbors are willing to help me when I am in need.
Mediating variable	Sleep quality	How would you rate your sleep quality in the past month?
Dependent variables	Subjective physical health	How do you feel about your current physical health?
		The extent to which you are unable to perform your expected work or daily activities due to health problems.
		In the past four weeks, how much has your illness affected your normal functioning?
	Objective physical health	Number of 12 common chronic diseases.
Control variables	Sample properties	Gender
		Perceived socioeconomic status
		Age

#### 2.2.1 Sleep quality

We employed a self-assessment survey to measure the dependent variable of our study, which is the comprehensive evaluation of sleep quality. This measure was recorded using a 4-point rating scale (“1 = very poor” to “4 = very good”), where higher scores corresponded to more favorable sleep quality experienced by the participants.

#### 2.2.2 Healthy community geographical environment

##### 2.2.2.1 Healthy environment

In CGSS 2021 survey, participants rated the severity of four environmental issues around their residential community: air pollution, water pollution, noise pollution, and insufficient lighting. These measures were recorded using a 4-point rating scale (“1 = very severe” to “4 = not severe at all”), where higher scores indicated a perception of a healthier community environment. The Cronbach’s *α* for scores from the healthy environment was 0.726.

##### 2.2.2.2 Healthy neighborhood

In the CGSS 2021 survey, participants provided ratings for safety, neighborly relations, and community assistance within their residential area. These measures were recorded using a 5-point rating scale (“1 = completely agree” to “5 = completely disagree”), which were reverse-scored during formal data analysis. Higher scores reflected a perception of healthier community neighborhood. The Cronbach’s *α* for scores from the healthy neighborhood was 0.713.

##### 2.2.2.3 Healthy service

In the CGSS 2021 survey, participants rated the services available in their residential area, including public facilities, sports and exercise amenities, and the availability of vegetables and fruits. These measures were recorded using a 5-point rating scale (“1 = completely agree” to “5 = completely disagree”), and were reverse-scored during formal data analysis. Higher scores indicated a perception of healthier community services. The Cronbach’s α for scores from the healthy neighborhood was 0.549.

#### 2.2.3 Physical health

In this study, physical health is divided into objective and subjective physical health.

##### 2.2.3.1 Objective physical health

In the CGSS 2021 survey, participants were asked whether they had any of the 12 common chronic diseases. Affirmative responses were scored as 1 for each disease, while negative responses were scored as 0. Subsequently, during formal data analysis, the total score for chronic diseases was reverse-scored. Higher scores indicated better objective physical health of the participants.

##### 2.2.3.2 Subjective physical health

In the CGSS 2021 survey, participants provided ratings on three aspects of their physical health perception. All three items were assessed using a 5-point scale, where higher scores indicated a healthier subjective physical condition. The Cronbach’s α for scores from the subjective physical health was 0.814.

#### 2.2.4 Control variables

This study incorporated control variables, including gender, age, and perceived socioeconomic status.

### 2.3 Data analysis

The analysis was conducted using SPSS 24.0 for descriptive statistics and Pearson correlation analysis. Subsequently, a structural equation modeling was performed using Amos 25.0 to examine the mediating role of sleep quality in the relationship between healthy community and physical health among older adults. To ensure the robustness of the mediation analysis, the bias-corrected bootstrap method with 5,000 resamples was employed to estimate the indirect effects. A mediation effect was considered statistically significant if the 95% confidence interval (CI) did not include zero.

To reduce common-method-bias due to self-report, this study controlled for it procedurally and statistically. In terms of procedures, CGSS2021 used anonymous surveys and reverse scoring of some items to carry out certain controls; in terms of statistics, we examined common method variance. Harman’s single factor test found that a total of 11 factors had eigenvalues greater than 1 and the first variance explanation rate was 11.528%, which is less than the critical value of 40%, indicating no significant common method bias in this study.

## 3 Results

### 3.1 Correlation analysis between variables

Pearson correlation analysis was conducted to explore the pairwise relationships among health environment, health services, healthy neighborhood, sleep quality, and subjective and objective physical health. The results revealed significant positive correlations between health environment and healthy neighborhood, sleep quality, and objective physical health. Furthermore, significant positive correlations were observed between health services and healthy neighborhood, sleep quality, and subjective physical health. Healthy neighborhood exhibited significant positive associations with sleep quality and subjective physical health. Sleep quality displayed a significant positive correlation with both subjective and objective physical health (see Table 3).

**Table 3:** Correlation results between variables.

	Healthy environment	Healthy service	Healthy neighborhood	Sleep quality	Subjective physical health
Healthy environment	1				
Healthy service	−0.042	1			
Healthy neighborhood	0.186^***^	0.291^***^	1		
Sleep quality	0.160^***^	0.137^***^	0.138^***^	1	
Subjective physical health	0.039	0.185^***^	0.109^***^	0.406^***^	1
Objective physical health	0.133^***^	−0.006	−0.020	0.199^***^	0.412^***^

^***^p < 0.001.

### 3.2 Mediation analysis

**Figure 3:**
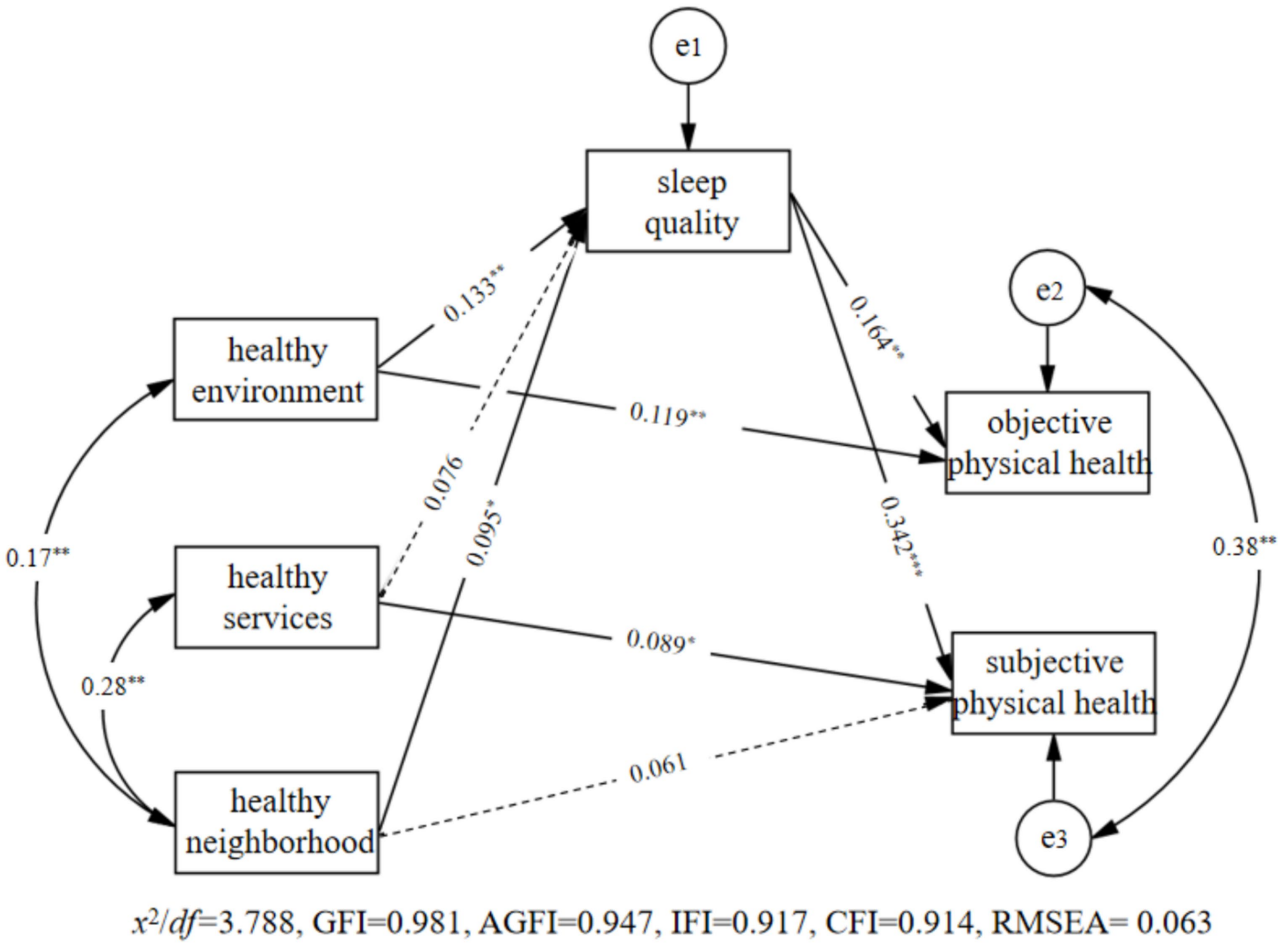
Mediation model of sleep quality.

Based on the results of the correlation analysis, we constructed the mediation model illustrated in Figure 3. During data analysis, this study also included gender, age, and perceived socioeconomic status as control variables in the model. We conducted analysis using Amos 24.0 to test the proposed hypothesis model.

Results, as shown in Figure 3, indicate that the model fits well. Firstly, the health environment significantly predicted sleep quality (*β* = 0.133, *p* = 0.002), and both health environment and sleep quality significantly predict objective physical health (*β =* 0.119, *p* = 0.004; *β* = 0.164, *p* = 0.001). Secondly, healthy service does not significantly predicted sleep quality (*β* = 0.076, *p* = 0.067), and both health services and sleep quality significantly predict subjective physical health (*β* = 0.089, *p* = 0.013; *β* = 0.342, *p* = 0.001). Lastly, health neighborhood significantly predicted sleep quality (*β* = 0.095, *p* = 0.025). Although health neighborhood does not significantly predict subjective physical health (*β* = 0.061, *p* = 0.070), sleep quality significantly predicts subjective physical health (*β* = 0.342, *p* = 0.001).

This study further used the deviation Bootstrap method to examine the mediating effect of sleep quality. From the original sample (*N* = 696), 2000 samples were randomly selected to estimate the indirect effect. If the 95% confidence interval (CI) does not contain 0, it means that the mediation effect is significant. The results found that: (1) The 95% CI of sleep quality in the relationship between healthy environment and objective physical health is [0.007, 0.043], excluding 0, indicating the mediating effect of sleep quality in the relationship between healthy environment and objective physical health is significant; (2) The 95% CI of the mediating effect of sleep quality on the relationship between healthy neighborhood and subjective physical health is [0.005, 0.064], excluding 0, indicating that the mediating effect of sleep quality on the relationship between healthy neighborhood and subjective physical health is significant.

## 4 Discussion

Our study explores the associations between multi-dimensional healthy community environments and older adults’ physical health, and examines the mediating role of sleep quality in the context of China. We found that different dimensions of healthy community environments contributed differently to various aspects of older adults’ physical health. We discuss our research findings in detail below.

Our study revealed a relationship between healthy environment and objective physical health in older adults, which partly supported hypothesis1A. We attribute this phenomenon to the following main reasons: Firstly, it is determined by the attributes of environmental pollution. A study has shown that the negative effects of pollution on the health levels of middle-aged and older adults increase over time (Ye and Huang, 2022). The impact of environmental pollution on physical health is long-term, and the environmental pollution in the communities where older adults reside silently affects their physical health status. Secondly, it is inherent to the characteristics of the aging process in the elderly. As older adults experience a decline in their physiological functions, their defense and immune systems become more vulnerable, making them more susceptible to health issues caused by environmental pollution. This, in turn, increases the risk and number of chronic diseases, ultimately leading to a decline in objective physical health. Interestingly, although a healthy environment was associated with better objective health, it showed no significant relationship with subjective health. This may be due to the background nature of environmental factors, which are less salient than interpersonal or service-related experiences. Subjective health is shaped by emotional feedback and cognitive attribution, and the preventive (rather than restorative) effects of the environment may go unnoticed.

Environmental exposure has cumulative physiological consequences, making its impact more readily captured in objective health outcomes. By contrast, the stronger association between healthy services and subjective health may reflect perceived accessibility to community resources, which can quickly shape individuals’ health perceptions. Our study found a correlation between healthy service and subjective physical health of older adults, which partly supported hypothesis1B. However, this result was inconsistent with the findings of Yuan et al. (2020). The data utilized in that study was collected in 2016, a period in which there might have been an imbalance in community development and public service provision in China. However, since 2016, China has continually initiated the “15-Minute Living Circle” initiative in communities nationwide (Zhang et al., 2023). The implementation of the “15-Minute Living Circle” initiative has contributed to an improvement in community health services, providing older adults with essential exercise facilities, convenient daily necessities, and expanded healthcare facilities. This multifaceted approach has effectively met the needs of older adults and subsequently enhanced their subjective perception of physical health. In communities with high service accessibility, older adults are more likely to maintain better physical health due to easier and more reliable access to essential health-related resources (Liu et al., 2024; Tani et al., 2018; Zhang et al., 2017). The availability of fresh food and healthcare services within a short distance reduces physical exertion and logistical barriers, making it easier for individuals to manage their health on a daily basis. Such convenience likely enhances residents’ sense of physical well-being and contributes to more positive self-rated health assessments.

A correlation between healthy neighborhood and the subjective physical health of older adults was supported in our study, which partly supported hypothesis1C. Positive social interactions can to some extent meet the relational needs of older adults. When these relational needs are met, individuals tend to experience reduced stress levels and an enhancement of their psychological well-being (Şahin et al., 2019). This improved psychological well-being effectively predicts an individual’s subjective physical health. This health-promoting mechanism may be especially pronounced in China’s collectivist cultural context, where social connectedness and interpersonal interdependence are highly valued. In such environments, older adults are more likely to derive psychological comfort and emotional security from strong neighborhood ties, making neighborhood cohesion a particularly potent factor influencing health. Additionally, when the community in which one resides fosters a greater sense of security, it generates positive emotional experiences. These positive emotional experiences contribute to the enhancement of the immune system’s functioning, which is particularly beneficial for older adults. Consequently, the positive emotional experiences triggered by a sense of security can, to a certain extent, promote the perception of higher levels of physical health.

In addition to the direct associations between healthy community environments and older adults’ physical health, our study also found two indirect pathways through which community factors influence health outcomes via sleep quality. First, this study identified that sleep quality plays a mediating role in the relationship between the healthy environment and the objective physical health of older adults, which partly supported hypothesis 2A. According to the environmental threat theory, various environmental pollutants act as stressors for individuals, increasing their stress levels (Xiang et al., 2017). When stress occurs, the hypothalamus releases corticotrophin-releasing hormone, initiating the pituitary and adrenal glands to secrete stress hormones, which keep individuals in an alert state, causing disruptions in the physiological and psychological mechanisms of sleep (Campbell et al., 2015). Poor sleep quality has been linked to elevated nighttime blood pressure, increased cardiovascular risk (Pepin et al., 2014; Kara and Tenekeci, 2017), impaired glucose regulation (Barone and Menna-Barreto, 2011), and a heightened incidence of falls among older adults (Min and Slattum, 2018). These biological consequences suggest that sleep serves as a crucial restorative process for immune function, energy replenishment, and physiological stability. When older adults experience chronic sleep deprivation or disturbances, they are more vulnerable to the onset and progression of health conditions, ultimately resulting in diminished objective physical health. Conversely, as sleep quality improves, individuals possess healthier organ systems, circulatory systems, and more, reducing the risk of older adults developing more chronic diseases.

Second, this study found that sleep quality mediates the relationship between healthy neighborhood and subjective physical health of older adults, which supported partly hypothesis 2C. According to the main effects of social support (Thoits, 1983), positive neighborhood interactions are a significant form of social support for older adults. Through continuous interactions, older adults obtain more relationships and support, which satisfy their relational needs, enhance positive emotional experiences, reduce stress, and improve sleep quality. In addition, a good sense of security will increase an individual’s positive emotional experience and reduce short-term worries and fears. A good emotional experience will reduce the release of individual stress hormones, thereby increasing sleep quality. As sleep quality continuously improving, sleep helps the body’s physiological functions, such as eliminating fatigue, restoring energy, and promoting recovery. Consequently, older adults subjectively perceive higher levels of physical health. Overall, our findings emphasize the importance of sleep quality as a psychological and physiological bridge linking the external community environment to internal health outcomes.

## 5 Theoretical and practical implications

Our study, grounded in the concept of “healthy community,” has constructed relationships and mechanisms among the quality of the healthy community environment, sleep quality, and physical health of older adults, providing fresh insights into this field. Our findings indicate that the perceived quality of the community environment plays a vital role in shaping both the objective and subjective physical health of older adults, with sleep quality serving as a key mediating factor. Theoretically, this study expands existing research on community health and aging by examining how perceived features of the community environment relate to both objective and subjective physical health outcomes in older adults. Specifically, the study explored the effects of three key dimensions of a healthy community—healthy environment, healthy service, and healthy neighborhood—on older adults’ physical health and further investigated the mediating role of sleep quality in these associations. These findings contribute to a more comprehensive theoretical framework that links external environmental conditions with internal physiological mechanisms affecting health, and provide a theoretical basis for understanding how community-level factors shape health trajectories in later life.

From a practical standpoint, this study provides targeted guidance for policymakers and urban planners aiming to promote healthy aging in community settings. First, improving environmental conditions (e.g., such as reducing air, water, and noise pollution, as well as ensuring adequate lighting) may significantly contribute to better physical health outcomes among older adults. These forms of environmental stress, if left unaddressed, may not only worsen physical health directly but also disrupt sleep, thereby elevating long-term health risks. Interventions focused on pollution control and environmental hygiene should be prioritized in urban aging policies. Second, strengthening healthy service provision is crucial to improving subjective physical health among older adults. This includes ensuring that the living environment supports physical activity, offers access to fresh fruits and vegetables, and provides adequate public facilities such as parks and community centers. Policymakers can implement this by advancing community infrastructure planning and ensuring these services are located within walkable distances, particularly in residential areas with a high density of older adults. Third, promoting a supportive and secure neighborhood atmosphere is essential. Elements such as neighborhood safety, mutual care, and reliable social assistance contribute to a sense of belonging and emotional stability among older adults. These positive social conditions have been shown in our study to enhance sleep quality and, consequently, improve individuals’ perceived health status. These recommendations align with China’s ongoing shift toward active social policy in response to population aging. As Zhu and Walker (2022) emphasize, promoting aging in place and investing in community-based services are central to building a sustainable aging strategy. Our findings offer empirical support for these priorities by highlighting how community-level environmental and social improvements can enhance older adults’ physical health.

Together, these findings underscore the importance of comprehensive, multi-dimensional interventions that address physical, infrastructural, and social aspects of the living environment to support the well-being of older adults. Altogether, the findings suggest that promoting healthy aging requires a multi-level approach that addresses the physical, service-related, and social environments of communities. Policymakers should consider adopting integrated environmental strategies guided by the socio-ecological model to build age-friendly communities that support the well-being of older adults.

## 6 Limitations and future perspectives

While this study has made some new findings, there are still several research limitations that need further refinement in future studies. Firstly, this study primarily relied on a subjective perspective to assess the perceived healthy community environment among older adults and explored its impact mechanisms on their physical health. Future research could consider employing techniques like web scraping to collect environmental quality data (such as air and water quality) and Geographic Information Systems to measure the accessibility and coverage of community services objectively. This approach would provide a comprehensive understanding of the healthy community environment and its components, shedding light on their impact mechanisms on the physical health of older adults from an objective perspective. Secondly, this study exclusively selected sleep quality as the mediating variable in the impact mechanism. Future research could explore alternative psychological variables, such as mental health, or different objective indicators like the Normalized Difference Vegetation Index, to identify mediating or moderating models within the impact mechanism. Third, the study did not account for potential unmeasured confounding variables, such as medication use, or diagnosed sleep disorders, which may influence both sleep quality and physical health. Although key demographic variables (gender, age, and perceived socioeconomic status) were statistically controlled, the lack of control for these health-related confounders may introduce bias in the mediation analysis. Future research is encouraged to collect relevant covariates and assess the robustness of mediation pathways accordingly. Lastly, due to the nesting of individual variables within communities, future research could continue to collect individual-community nested data and employ multilevel linear mixed models to investigate the impact mechanisms on the physical health of older adults, validating the conclusions drawn in this study.

## 7 Conclusion

Sleep quality played a mediating role in the relationship between the healthy community environment and the physical health of older adults. Specifically, it functions as a mediator in two contexts: (1) Sleep quality acted as a mediator in the relationship between the healthy environment and objective physical health of older adults; (2) Sleep quality served as a mediator in the relationship between healthy neighborhood and subjective physical health of older adults.

## Data Availability

Publicly available datasets were analyzed in this study. This data can be found here: http://www.cnsda.org.

## Footnotes

^1^ http://cgss.ruc.edu.cn/
